# *Pseudomonas chlororaphis* A54 Enhances Drought Tolerance in *Pinus sylvestris* var. *mongolica* Through Coordinated Plant Physiological, Rhizosphere Microbial, and Soil Functional Responses

**DOI:** 10.3390/plants15101503

**Published:** 2026-05-14

**Authors:** Qian Song, Xiaoshuang Song, Xun Deng

**Affiliations:** 1 State Key Laboratory of Plateau Ecology and Agriculture, Qinghai University, Xining 810016, China; 2023990003@qhu.edu.cn; 2 Institute of Forestry Protection, Heilongjiang Forestry Academy, Harbin 150040, China

**Keywords:** *Pinus sylvestris* var. *mongolica*, drought stress, plant growth-promoting rhizobacteria, rhizosphere assembly, soil function, oxidative stress, nutrient maintenance, genome-based functional potential

## Abstract

Drought severely restricts the growth and establishment of *Pinus sylvestris* var. *mongolica* seedlings, whereas the mechanisms by which plant growth-promoting rhizobacteria improve host drought tolerance remain incompletely understood. In this study, strain A54 was evaluated under four drought gradients (ND, LD, MD, and SD) in a greenhouse pot experiment. Seedling growth, nutrient accumulation, physiological traits, rhizosphere bacterial communities, soil functional variables, genome annotation, and qRT-PCR were integrated to clarify the drought-alleviating effects of A54. At the strain level, A54 maintained growth and ACC deaminase-associated functional performance under PEG-induced osmotic stress. A54 inoculation alleviated drought-induced growth suppression, with seedling height increasing by 69.7% under MD and 87.7% under SD relative to the corresponding controls. A54 also improved nutrient maintenance, especially Stem TN and Leaf TK, enhanced antioxidant capacity, and reduced osmotic stress, membrane lipid peroxidation, and stress-hormone accumulation. In the rhizosphere, A54 reshaped bacterial community structure by increasing the proportion of persistent taxa and selectively enriching drought-associated taxa, especially *Pseudarthrobacter*. A54-treated soils also maintained higher levels of available nutrients and enzyme activities under drought. Genome annotation and representative gene expression further supported the functional potential of A54 in nitrogen metabolism, ACC deaminase-associated ethylene regulation, oxidative defense, and osmotic or ion homeostasis. supporting its role in enhancing drought tolerance. These findings support the potential application of A54 as a bio-inoculant to improve afforestation performance under water-limited conditions.

## 1. Introduction

Drought is one of the most important abiotic stresses limiting plant growth, seedling establishment, and ecosystem restoration in water-limited regions [1]. Its effects extend from cellular metabolism to vegetation regeneration, and these constraints are expected to intensify under increasingly unstable climatic conditions [2]. *Pinus sylvestris* var. *mongolica* is an important afforestation species in northern China because of its ecological adaptability and windbreak value, but its seedling establishment and early growth remain highly sensitive to soil water deficit [3]. Improving drought tolerance in this species is therefore important for both plantation stability and ecological restoration in arid and semi-arid regions [4].

Plant growth-promoting rhizobacteria (PGPR) have attracted increasing attention as environmentally friendly tools for improving plant performance under drought stress [5]. Previous studies have shown that beneficial rhizobacteria can enhance drought tolerance by improving nutrient acquisition, regulating phytohormone balance, reinforcing antioxidant protection, and modifying rhizosphere microbial communities [6]. Recent microbiome studies suggest that plant drought responses involve coordinated changes in the host, the soil environment, and the rhizosphere microbiome, rather than as changes in host traits alone [7]. This perspective has shifted the focus from isolated physiological indicators to integrated plant–soil–microbe interactions under drought [8].

However, several important questions remain unresolved. First, most PGPR-mediated drought studies have focused on crops, whereas conifer seedlings in afforestation systems have received far less attention [9]. Second, many studies have emphasized either host physiological regulation or rhizosphere microbial shifts, but fewer have connected plant nutrient status, soil functional variables, microbial assembly, and bacterial functional traits within the same framework [10]. In addition, current studies differ in how PGPR-mediated drought resistance is interpreted. Some evidence suggests that microbial inoculation acts mainly through direct regulation of host physiology, whereas other studies indicate that selective rhizosphere assembly and the maintenance of functionally important taxa may be more central to stress alleviation [11]. It also remains unclear whether beneficial inoculation primarily increases overall microbial diversity or instead promotes a more persistent and functionally coordinated community structure [12]. However, few studies have simultaneously linked host nutrient status, soil function, rhizosphere assembly, and bacterial functional potential within the same framework in conifer seedlings under drought stress.

In this study, we investigated the effects of strain A54 on *P. sylvestris* var. *mongolica* seedlings under different drought gradients in a greenhouse pot experiment. We integrated analyses of seedling growth, plant nutrient accumulation, physiological traits, rhizosphere bacterial communities, soil functional variables, genome-based functional potential, and representative gene expression to determine whether A54 enhances drought tolerance through coordinated regulation across the plant, soil, and microbial compartments. Based on the physiological and ecological roles of plant growth-promoting rhizobacteria under water limitation, we hypothesized that A54 would alleviate drought-induced growth inhibition by improving nutrient maintenance, antioxidant defense, and osmotic regulation, restructure the rhizosphere bacterial community under drought by increasing the proportion of persistent taxa and selectively enriching drought-associated taxa, and exhibit functional traits associated with drought adaptation, particularly those related to nitrogen metabolism, ACC deaminase-associated ethylene regulation, oxidative defense, and osmotic or ion homeostasis.

## 2. Results

### 2.1. In Vitro Drought Tolerance and ACC Deaminase Activity of Strain A54

As shown in Supplementary Figure S1, strain A54 showed sustained growth across the tested osmotic stress levels, although growth was progressively reduced as water potential decreased. Under PEG 0 and PEG −0.05, protein concentration increased during the early incubation period and remained at relatively high levels before declining at later stages, whereas under PEG −0.30, the overall growth level was markedly lower than that under the two lower-stress treatments, although a transient increase was still observed at the middle stage. In addition, ACC deaminase activity decreased gradually with increasing water stress, but remained detectable even under PEG −0.3. These results indicate that A54 possesses substantial tolerance to osmotic stress and retains ACC deaminase-associated stress-response potential under drought-related conditions.

Strain A54 exhibited multiple plant growth-promoting traits in vitro (Supplementary Table S1). It showed nitrogen-fixation ability, phosphorus-solubilizing capacity, and IAA production, and was also positive for siderophore, HCN, pectinase, chitinase, cellulase, and protease production. These traits indicate that A54 has broad functional potential related to nutrient acquisition and growth promotion.

### 2.2. Plant Nutrient Accumulation Under Drought and A54 Inoculation

**Figure 1:**
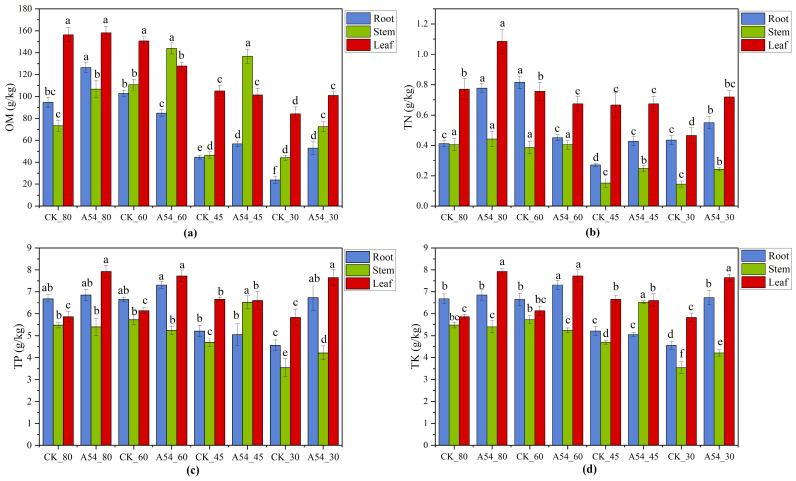
Effects of A54 inoculation on plant nutrient accumulation in *P. sylvestris* var. *mongolica* under different drought gradients. (**a**) Organic matter (OM), (**b**) total nitrogen (TN), (**c**) total phosphorus (TP), (**d**) total potassium (TK). CK, uninoculated control; A54, seedlings inoculated with strain A54. Different lowercase letters indicate significant differences among treatments at *p* < 0.05.

As shown in Figure 1, drought altered nutrient accumulation patterns in roots, stems, and leaves, and the responses to A54 inoculation were strongly tissue- and gradient-dependent. Overall, severe drought was accompanied by reduced OM, TN, TP, and TK in most tissues, whereas A54 partially alleviated these declines and improved nutrient maintenance under water deficit.

For OM, A54 generally increased nutrient accumulation under ND and SD, and also markedly improved root and stem OM under MD. TN showed a clearer recovery under stronger drought, with A54 enhancing TN accumulation in roots and stems under MD and SD and partially restoring leaf TN under SD. For phosphorus and potassium, the response was more tissue-specific. Leaf TP and especially Leaf TK were generally higher in A54-inoculated seedlings than in the corresponding controls, whereas root and stem responses varied with drought level. Notably, A54 promoted TK accumulation in leaves across all drought gradients and improved root TK under drought conditions.

A54 most consistently improved nutrient maintenance in Stem TN and Leaf TK, with the clearest effects observed under moderate and severe drought conditions. Taken together, these results indicate that A54 did not uniformly increase all nutrient pools, but helped maintain tissue nutrient status under drought through selective regulation of nutrient accumulation and distribution, thereby providing nutritional support for the improved growth and physiological performance of inoculated seedlings.

### 2.3. A54 Alleviated Drought-Induced Growth Inhibition in Pinus sylvestris var. mongolica Seedlings

As shown in Table 1, increasing drought intensity progressively suppressed seedling growth. Compared with the corresponding control, A54 inoculation consistently improved seedling height and biomass, with stronger effects under moderate and severe drought. Under MD, A54 increased seedling height, diameter, shoot dry weight, and root dry weight by 69.7%, 28.9%, 64.7%, and 25.0%, respectively, whereas under SD the corresponding increases reached 87.7%, 38.5%, 42.9%, and 66.7%. Notably, seedling height under SD was 1.88-fold that of the control, indicating that A54 markedly alleviated drought-induced growth suppression.

### 2.4. Oxidative, Osmotic, and Hormonal Responses Under Drought and A54 Inoculation

**Figure 2:**
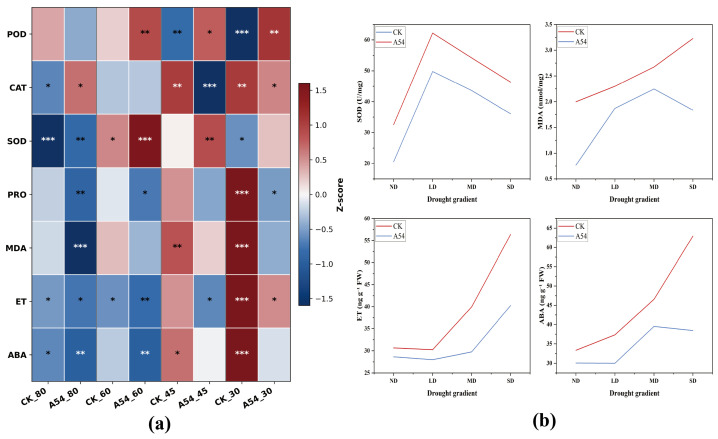
Effects of A54 inoculation on oxidative, osmotic, and hormonal responses of *P. sylvestris* var. *mongolica* seedlings under different drought gradients. (**a**) Heatmap of physiological indicators, including POD, CAT, SOD, PRO, MDA, ET, and ABA, under different drought gradients with or without A54 inoculation. (**b**) Line plots showing the changes in SOD activity, MDA content, ET and ABA contents across drought gradients. ND, normal water supply; LD, light drought; MD, moderate drought; SD, severe drought. CK, uninoculated control; A54, seedlings inoculated with strain A54. Values in the heatmap were standardized by column using Z-score transformation before visualization. To simplify comparison of relative response intensity among indicators, asterisks are used to denote the magnitude of standardized deviation from the mean, where *, **, and *** represent increasing absolute deviation.

As shown in Figure 2, A54 inoculation induced coordinated changes in oxidative, osmotic, and hormonal indicators across drought gradients. The heatmap revealed that the physiological response of inoculated seedlings was characterized mainly by enhanced antioxidant capacity and reduced accumulation of stress-related metabolites and hormones rather than by a uniform increase in all measured traits.

Among the antioxidant enzymes, SOD showed the clearest response to A54. Compared with the corresponding controls, SOD activity was consistently higher in A54-inoculated seedlings across all drought gradients, with the strongest increase under LD and MD. POD also tended to increase under LD, MD, and SD, whereas CAT showed a drought-gradient-dependent response and was not consistently enhanced by A54. These results suggest that A54 primarily strengthened antioxidant defense through selective activation of specific enzymes rather than uniformly stimulating the entire antioxidant system.

In contrast, the osmotic stress marker PRO and the membrane lipid peroxidation product MDA generally accumulated under drought, especially in the uninoculated controls. A54 markedly suppressed this accumulation, and the difference became more evident under stronger drought stress. Likewise, both ET and ABA increased progressively with drought intensity in the control seedlings, particularly under SD, whereas A54-treated seedlings maintained substantially lower levels. Together, these results indicate that A54 alleviated drought-induced physiological stress mainly by enhancing antioxidant defense while restraining osmotic imbalance, oxidative damage, and stress-hormone accumulation.

To further evaluate the effects of A54 inoculation, drought gradients, and their interaction on physiological traits, the corresponding ANOVA results are provided in Supplementary Table S2. Drought gradients significantly affected most measured parameters, whereas under LD no significant effects were observed for CAT, POD, PRO, or ET. The combined effects of A54 inoculation and drought gradients were significant for most physiological traits, except that the A54 × LD interaction was not significant for ET.

### 2.5. A54 Inoculation Reshaped Rhizosphere Bacterial Community Structure Across Drought Gradients

**Figure 3:**
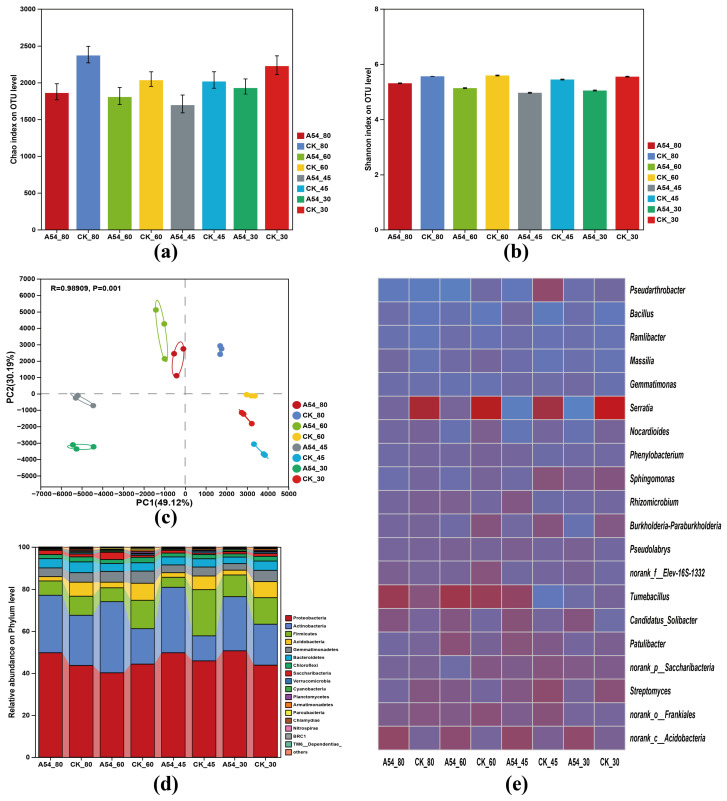
Effects of A54 inoculation on rhizosphere bacterial community structure under different drought gradients. (**a**) Chao richness, (**b**) Shannon diversity, (**c**) principal component analysis (PCA) plot. The treatment groups correspond to CK and A54 under ND, LD, MD, and SD conditions, where CK indicates the uninoculated control and A54 indicates seedlings inoculated with strain A54, (**d**) relative abundance of dominant bacterial phyla, and (**e**) heatmap of representative bacterial genera under different drought gradients with or without A54 inoculation. CK, uninoculated control; A54, seedlings inoculated with strain A54.

Coverage values remained high and comparable among treatments, indicating sufficient sequencing depth; detailed values are provided in Supplementary Table S3. As shown in Figure 3a,b, the alpha-diversity indices varied among treatments. Compared with the corresponding controls, A54-inoculated samples generally exhibited lower Chao richness and Shannon diversity, indicating that A54 did not increase overall bacterial richness or diversity, but instead may have promoted a more selective assembly of the rhizosphere community.

A54 inoculation generally reduced Chao richness and Shannon diversity, suggesting that the rhizosphere community became more selective under inoculation, with assembly shifting toward a narrower but potentially more drought-adapted bacterial composition.

Principal component analysis revealed clear separation among treatments (Figure 3c). PC1 and PC2 explained 49.12% and 30.19% of the variation, respectively, and ANOSIM further confirmed that bacterial community composition differed significantly among treatments (R = 0.98909, *p* = 0.001). Notably, A54 and CK samples were clearly separated within the same drought gradient, suggesting that inoculation strongly altered rhizosphere bacterial community structure under different water conditions.

At the phylum level, the rhizosphere bacterial communities were mainly dominated by Proteobacteria and Actinobacteria, followed by Firmicutes, Acidobacteria, and Gemmatimonadetes (Figure 3d). Although the dominant phyla were shared among treatments, their relative abundances varied across drought gradients and inoculation treatments, indicating that both drought intensity and A54 inoculation contributed to shifts in community composition.

At the genus level, the heatmap further showed distinct treatment-dependent changes in representative taxa (Figure 3e). Several genera, including *Pseudarthrobacter*, *Bacillus*, *Serratia*, *Streptomyces*, and *Pseudolabrys*, displayed different abundance patterns across drought gradients and inoculation treatments, suggesting that A54 selectively modulated drought-associated rhizosphere bacteria. Together, these results indicate that A54 reshaped rhizosphere bacterial communities mainly through selective compositional reassembly rather than by increasing overall diversity.

### 2.6. A54 Increased Community Persistence and Selectively Modulated Keystone Candidates

**Figure 4:**
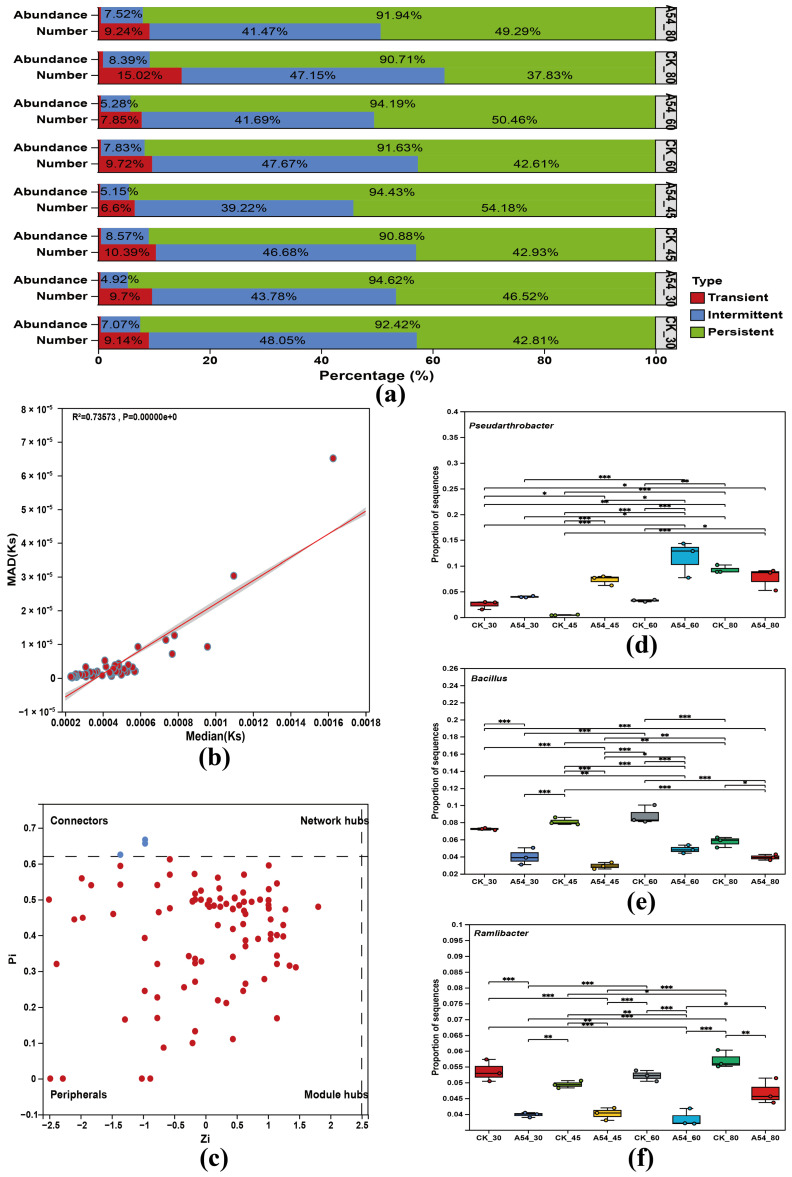
Effects of A54 inoculation on community persistence and keystone candidates under different drought gradients. (**a**) Proportions of transient, intermittent, and persistent taxa in terms of OTU number and relative abundance; (**b**) linear regression between Median (Ks) and MAD (Ks); (**c**) Zi–Pi plot showing topological roles of taxa in the co-occurrence network. Zi–Pi analysis was used to classify node roles in the co-occurrence network based on topological features. According to the standard thresholds, taxa were classified as module hubs (Zi > 2.5 and Pi < 0.62), connectors (Zi < 2.5 and Pi > 0.62), network hubs (Zi > 2.5 and Pi > 0.62), and peripherals (Zi < 2.5 and Pi < 0.62), blue dots indicate connectors, whereas red dots indicate peripherals. No module hubs or network hubs were detected; and (**d**–**f**) relative abundance of representative keystone candidates under different drought gradients with or without A54 inoculation. CK, uninoculated control; A54, seedlings inoculated with strain A54. In panels (**d**–**f**), asterisks indicate significant differences between CK and A54 within the same drought gradient: *, *p* < 0.05; **, *p* < 0.01; ***, *p* < 0.001.

As shown in Figure 4a, persistent taxa contributed more than 90% of the total community abundance in all treatments, whereas transient taxa accounted for only a small fraction. In terms of OTU number, persistent and intermittent taxa together constituted the major part of the community. Compared with the corresponding controls, A54 inoculation generally increased the proportion of persistent taxa and reduced the proportion of transient taxa, especially under LD and MD. Specifically, the numerical proportion of persistent taxa increased from 37.83% to 49.29% under ND, from 42.61% to 50.46% under LD, and from 42.93% to 54.18% under MD. These results suggest that A54 promoted a more persistent and less environmentally sensitive rhizosphere bacterial assemblage.

As shown in Figure 4b, Median(Ks) was positively correlated with MAD(Ks) (R^2^ = 0.73573, *p* < 0.001), indicating that taxa with low structural keystoneness were generally unlikely to act as keystone taxa, whereas taxa with higher keystoneness tended to show stronger variation among communities. This pattern suggests that keystone potential was context-dependent rather than uniformly stable across treatments.

Zi–Pi analysis showed that most taxa were classified as peripherals, whereas only a few were identified as connectors, and no module hubs or network hubs were detected (Figure 4c). This indicates that the topological importance of key taxa in the present network was mainly reflected by inter-module linkage rather than strong within-module dominance.

The abundance patterns of representative keystone candidates further demonstrated selective modulation by A54 (Figure 4d–f). *Pseudarthrobacter* was enriched by A54 under drought, particularly under LD and MD, whereas *Bacillus* and *Ramlibacter* generally showed lower relative abundance in A54-treated samples than in the corresponding controls. Together, these results indicate that A54 did not uniformly increase all key taxa, but instead reshaped the rhizosphere microbiome by enhancing community persistence and selectively regulating specific keystone candidates.

### 2.7. Soil Functional Patterns and Environmental Associations

**Figure 5:**
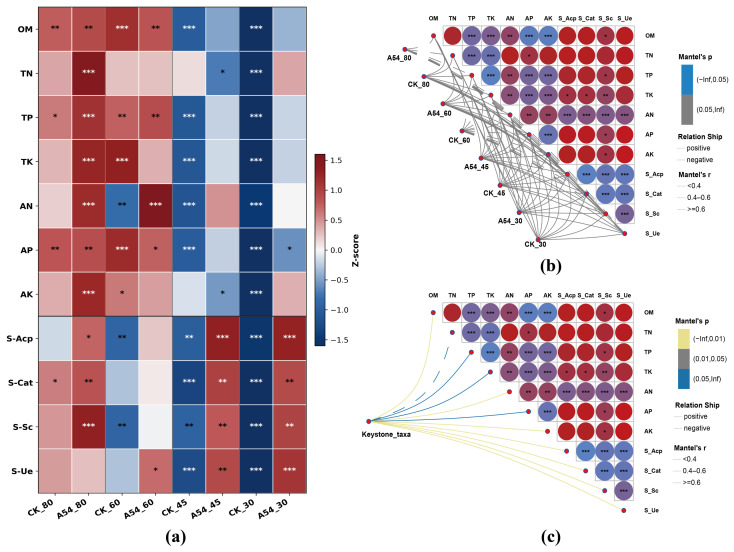
Effects of A54 inoculation on soil functional patterns and environmental associations under different drought gradients. (**a**) Heatmap of soil physicochemical properties and enzyme activities, Values in the heatmap were standardized by column using Z-score transformation before visualization. (**b**) Mantel test heatmap showing associations between soil environmental factors and the overall rhizosphere bacterial community, and (**c**) Mantel test heatmap showing associations between soil environmental factors and keystone taxa under different drought gradients with or without A54 inoculation. CK, uninoculated control; A54, seedlings inoculated with strain A54. Asterisks in panel (**a**–**c**) indicate relative response levels based on standardized values, where *, **, and *** represent increasing absolute deviation from the mean.

As shown in Figure 5a, soil physicochemical properties and enzyme activities exhibited clear treatment-dependent patterns across drought gradients. Relative to the corresponding controls, A54 inoculation generally maintained higher standardized levels of several soil variables under drought, whereas CK_SD showed the lowest values for most nutrient and enzyme indices. This trend was particularly evident for soil enzyme activities. Under MD and SD, A54-treated soils displayed markedly higher relative levels of S-Acp, S-Cat, S-Sc, and S-Ue than the corresponding controls, indicating that A54 helped maintain soil functional activity under water deficit. In addition, several nutrient-related variables, especially TN, AN, and AK, were partially restored in A54-treated soils under severe drought, suggesting that A54 improved the rhizosphere soil environment in a drought-dependent manner.

Mantel analysis further showed that variation in the overall soil bacterial community was associated with multiple soil environmental factors (Figure 5b). These associations were not isolated, because the embedded correlation matrix indicated coordinated relationships among soil nutrient variables and enzyme activities. More importantly, the keystone taxa displayed broad and relatively stronger associations with soil factors than did the community pattern alone (Figure 5c). In particular, available nutrient fractions and soil enzyme activities, including AN, AP, AK, S-Acp, S-Cat, S-Sc, and S-Ue, showed closer links with keystone taxa. Together, these results suggest that A54 did not merely alter individual soil variables, but reshaped a soil functional environment that was closely coupled with both community variation and the selective response of keystone taxa. Detailed results of the Monte Carlo test are provided in Supplementary Table S4.

### 2.8. Genome-Based Functional Potential and Expression of Representative Genes in Strain A54 Under Drought Stress

Genome assembly and annotation indicated that strain A54 had a 6.49 Mb genome with a GC content of 56.97% and 5745 predicted genes, providing a basis for subsequent functional analysis. Further annotation identified multiple genes potentially related to plant growth promotion and drought adaptation, including those associated with nitrogen metabolism, ACC deaminase/ethylene modulation, IAA-related traits, oxidative stress defense, and osmotic or ion homeostasis. Detailed genome assembly and annotation statistics are shown in Supplementary Table S5, and the full list of annotated genes potentially related to plant growth promotion and drought adaptation is provided in Supplementary Table S6.

**Figure 6:**
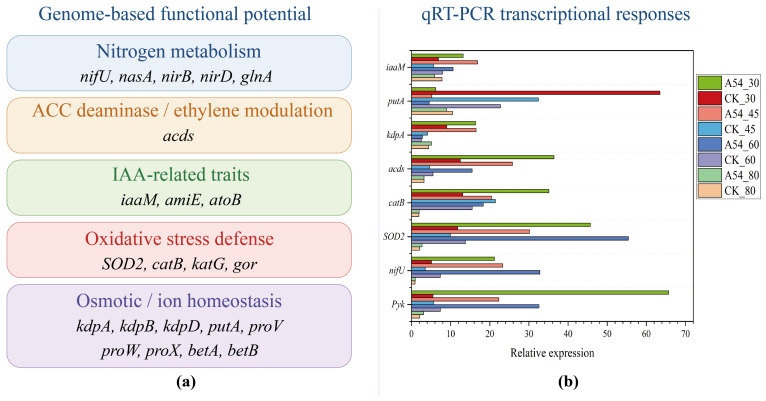
Genome-based functional potential and expression of representative genes in strain A54 under drought stress. (**a**) Genome-based functional modules potentially associated with plant growth promotion and drought adaptation in strain A54, including nitrogen metabolism, ACC deaminase/ethylene modulation, IAA-related traits, oxidative stress defense, and osmotic/ion homeostasis. (**b**) qRT-PCR expression of representative genes (pyk, nifU, SOD2, catB, acds, kdpA, putA, and iaaM) under different drought gradients. Relative expression values are shown for each gene across treatments.

Figure 6 showed that strain A54 possessed multiple genome-encoded functional modules related to plant growth promotion and drought adaptation, including nitrogen metabolism, ACC deaminase/ethylene modulation, IAA-related traits, oxidative stress defense, and osmotic/ion homeostasis. qRT-PCR further demonstrated that representative genes within these modules responded differentially across drought gradients, with *pyk*, *nifU*, *SOD2*, *catB*, *acds*, and *kdpA* showing relatively stronger induction under stress-associated conditions, whereas *putA* displayed a distinct pattern. These results provide molecular support for the drought-alleviating function of A54. Representative genes showed relatively stronger induction under drought treatment, as shown in Figure 6b.

### 2.9. Associations of Plant Nutrients with Soil Function and Key Bacterial Taxa Under Drought and A54 Inoculation

**Figure 7:**
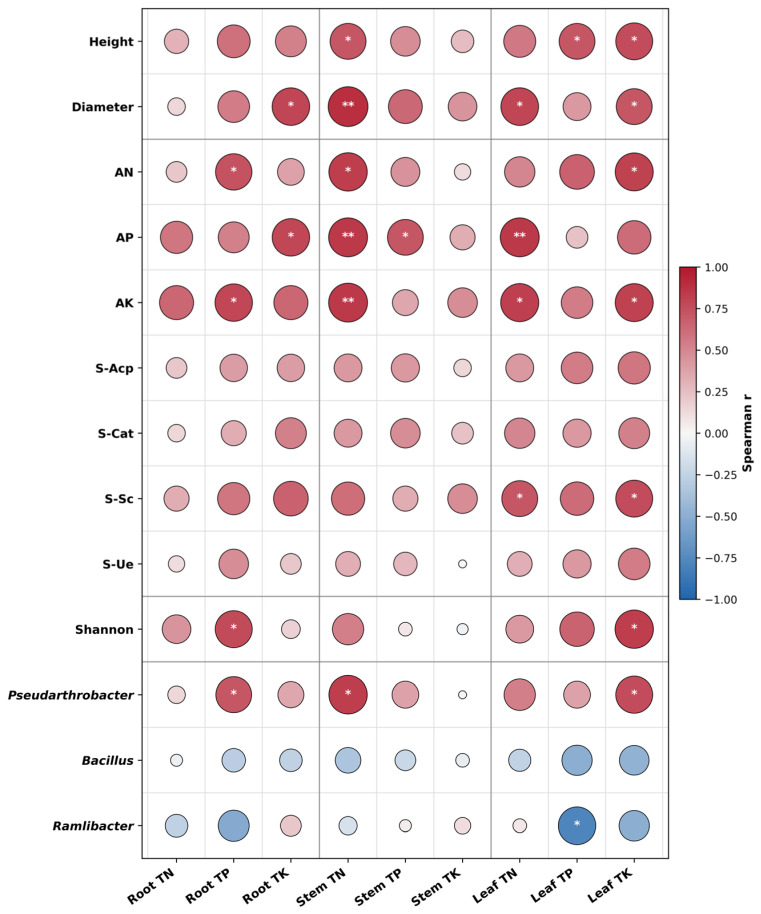
Spearman correlations of plant nutrient status with growth, soil function, and key bacterial taxa under drought and A54 inoculation. Asterisks indicate significant Spearman correlations: *, *p* < 0.05; **, *p* < 0.01.

As shown in Figure 7, plant nutrient status was closely associated with seedling growth, soil functional variables, and key bacterial taxa. Among the measured nutrient traits, Stem TN and Leaf TK exhibited the broadest and strongest positive correlations, suggesting that they were central nutrient variables linking host performance with the rhizosphere environment.

With respect to plant growth, Leaf TK was positively correlated with seedling height, diameter, and several biomass-related traits, whereas Stem TN also showed strong positive associations with height and diameter. These results indicate that improved nutrient maintenance, particularly in stem nitrogen and leaf potassium, was closely related to the growth-promoting effect of A54 under drought.

For soil variables, available nutrients and enzyme activities were more strongly associated with plant nutrient status than bulk variables. In particular, AN, AP, AK, and S-Sc showed relatively strong positive correlations with several nutrient traits, especially Stem TN, Leaf TN, and Leaf TK, indicating that the improvement in plant nutrient accumulation under A54 inoculation was tightly coupled with changes in rhizosphere nutrient availability and soil functional activity.

Among the microbial variables, *Pseudarthrobacter* showed clear positive correlations with several nutrient traits, especially Root TP, Stem TN, and Leaf TK, whereas *Ramlibacter* was negatively correlated with Leaf TP. By contrast, correlations involving *Bacillus* were generally weak and not significant. In addition, the Shannon index was positively associated with Root TP and Leaf TK, suggesting that nutrient maintenance under A54 inoculation was linked not only to individual keystone candidates but also to overall bacterial diversity.

Taken together, these results suggest that A54-mediated drought alleviation was associated with coordinated changes in plant nutrient accumulation, rhizosphere soil function, and selective bacterial responses, with Stem TN and Leaf TK emerging as key nutrient hubs in this integrated response network.

## 3. Discussion

### 3.1. A Multi-Level Framework for A54-Mediated Drought Alleviation

Drought is one of the major environmental constraints limiting the growth and establishment of afforestation tree species, and its effects on *Pinus sylvestris* var. *mongolica* seedlings are especially pronounced in water-limited regions [3,4,9]. Although plant growth-promoting rhizobacteria have been widely reported to alleviate drought stress, many previous studies have mainly focused on a single layer, such as host physiology or rhizosphere community change. In contrast, less attention has been paid to the links between host performance, soil function, rhizosphere assembly, and bacterial functional potential within the same framework [13,14]. In the present study, A54 consistently mitigated drought-induced growth suppression in *P. sylvestris* var. *mongolica* seedlings, and this effect was accompanied by coordinated shifts in tissue nutrient accumulation, physiological status, rhizosphere bacterial assembly, soil functional variables, and representative bacterial gene expression. These results indicate that the beneficial effect of A54 was not driven by a single process, but by coupled changes across the plant, soil, and microbial compartments. The main novelty of this study therefore lies not simply in identifying a beneficial strain, but in establishing a multi-level explanatory framework for A54-mediated drought alleviation under a unified plant–soil–microbe context.

### 3.2. Strain-Level Osmotic Stress Tolerance and Host Growth Buffering

The first line of evidence came from the intrinsic characteristics of the strain itself. Supplementary Figure S1 shows that A54 maintained growth under reduced water potential and retained detectable ACC deaminase activity even under stronger osmotic stress. This indicates that A54 itself has substantial osmotic stress tolerance and can preserve a key stress-related function under drought-related conditions. ACC deaminase can reduce excessive ethylene accumulation in plants under stress and thereby alleviate drought-induced growth inhibition, and this mechanism has been widely recognized as one of the major drought-alleviating traits of PGPR [15,16]. Therefore, Supplementary Figure S1 should not be regarded as simple background information for the strain. Instead, it shows that A54 already possessed the capacity to tolerate low water potential and maintain functional output before entering the rhizosphere, which provides a reasonable basis for the beneficial effects observed later in the pot experiment. This pre-adaptive capacity may be important for the persistence and functional performance of A54 under water-limited conditions, thereby supporting its potential application in drought-prone afforestation systems.

This strain-level capacity was directly reflected in the host phenotype. Table 1 showed that A54 increased seedling height and biomass across different drought gradients, and the promoting effect became more evident under moderate and severe drought. Similar patterns have been reported in previous PGPR studies, in which microbial buffering effects are often more apparent under stronger stress because microbial regulation of nutrient acquisition, hormonal balance, and oxidative protection becomes more functionally important at that stage [17]. Although PGPR-mediated drought alleviation has been reported in woody plants and afforestation systems, direct evidence specifically linking *Pseudomonas chlororaphis* to improved drought resistance in pine seedlings remains limited. The present study, therefore, provides additional evidence supporting the potential relevance of this genus in conifer drought adaptation under inoculation conditions. This point is important because it suggests that the role of A54 was not merely to promote growth under favorable conditions, but to reduce the magnitude of growth loss caused by drought. From the perspective of stress resistance, this buffering effect is more meaningful than simple growth stimulation, because it is more closely aligned with the practical goal of microbial application in afforestation under water-limited conditions [18].

### 3.3. Nutrient Maintenance and Physiological Regulation Under Drought

Figure 1 further shows that the effect of A54 on plant nutrient status was strongly tissue-dependent. OM, TN, TP, and TK in roots, stems, and leaves did not respond uniformly to inoculation, indicating that A54 did not simply increase all nutrient pools in all tissues, but selectively maintained particular nutrient traits. The correlation results further showed that Stem TN and Leaf TK had broader and more stable associations with growth, soil function, and key bacterial taxa. This result adds mechanistic depth to the effect of A54. Nitrogen in stem tissues is closely related to metabolic continuity and nutrient transport, whereas potassium in leaves is directly involved in osmotic adjustment, stomatal regulation, and assimilate transport, all of which are particularly important under drought stress [19]. Therefore, the drought-alleviating effect of A54 likely did not depend on a general increase in all nutrient pools, but rather on the preferential maintenance of nutrient traits with stronger functional relevance. This interpretation is more specific than the general statement that PGPR improves nutrient availability, and it also explains more effectively why inoculated seedlings maintained better growth under drought [20]. Nitrogen in stem tissues is closely related to metabolic continuity and nutrient transport, whereas potassium in leaves is directly involved in osmotic adjustment, stomatal regulation, and assimilate transport, all of which are particularly important under drought stress. In terms of novelty, this study did not treat nutrient variation as a secondary outcome, but further identified Stem TN and Leaf TK as two important nutrient nodes linking host performance with the rhizosphere environment, which has rarely been explicitly addressed in comparable PGPR drought studies.

Figure 2 shows that A54 significantly altered oxidative, osmotic, and hormonal responses under drought. SOD activity in A54-treated seedlings was consistently higher than that in the corresponding controls across drought gradients. POD also showed an overall increase under most drought conditions, whereas PRO, MDA, ET, and ABA were generally lower than those in the corresponding controls. Increased SOD and POD usually indicate stronger ROS-scavenging capacity, reduced MDA reflects lower membrane lipid peroxidation, and lower ABA and ET suggest a reduced stress-signaling burden [21,22,23]. What matters most here is not the change in any single indicator, but the overall consistency in direction. A54 did not push the host into a stronger stress state. Instead, it helped the host maintain a physiological state with lower damage and greater metabolic stability under drought. Combined with the retained ACC deaminase activity observed in Supplementary Figure S1, the lower ET level in A54-treated seedlings is consistent with a possible role of A54 in modulating ethylene-related stress responses. However, this result should be regarded as indirect evidence rather than direct proof of ACC deaminase-mediated regulation of ethylene accumulation in planta [24]. Unlike studies that explain PGPR effects mainly by increased antioxidant enzyme activity, the present work further showed that the suppression of PRO, MDA, ET, and ABA was equally important, suggesting that A54 reduced the actual stress burden on the host rather than simply amplifying defense signaling.

### 3.4. Rhizosphere Community Reassembly and Keystone Taxa Under Inoculation

At the rhizosphere level, Figure 3 indicates that the microbial effect of A54 was expressed mainly as structural reassembly rather than a simple increase in diversity. PCA clearly separated A54-treated samples from their corresponding controls, indicating a strong inoculation effect on community composition, whereas alpha diversity did not show a consistent increase. This means that the microbial effect of A54 was not a non-specific enrichment of richness, but a directed adjustment of rhizosphere composition. Under drought, this type of response is ecologically more meaningful than a simple diversity increase, because microbiome function often depends more on selective recruitment and community organization than on richness alone [25]. In terms of innovation, the present study did not stop at the common conclusion that inoculation altered community structure, but further linked this structural shift with persistent taxa, key bacterial taxa, and soil functional changes, thereby providing a more explicit functional interpretation of rhizosphere reassembly [14].

Figure 4 provides deeper evidence for this selective reassembly. A54 increased the proportion of persistent taxa and reduced the proportion of transient taxa, especially under LD and MD, indicating that it promoted a rhizosphere community with greater persistence and lower environmental sensitivity. At the same time, the keystone analysis suggested that the ecological importance of candidate taxa was context dependent, and Zi–Pi analysis showed that topologically important taxa were mainly connectors rather than module hubs or network hubs. This indicates that, in the present system, the role of key taxa was expressed mainly through linking different modules rather than occupying a dominant position within a single module. Such a network structure is consistent with the view that rhizosphere systems under drought may rely more on flexible coordination among modules than on a few highly centralized nodes [26]. In addition, A54 did not promote all representative taxa simultaneously, but selectively enriched those more closely linked to nutrient maintenance and drought-associated functions. *Pseudarthrobacter* was enriched under drought and positively correlated with Root TP, Stem TN, and Leaf TK; *Ramlibacter* was negatively correlated with Leaf TP; and *Bacillus* showed comparatively weaker associations with nutrient traits. The enrichment of *Pseudarthrobacter* may therefore be functionally meaningful in this system, because members of this genus have been reported in previous studies to be associated with stress tolerance, nutrient transformation, and other plant growth-promoting traits. Although the present study does not directly verify the functional contribution of *Pseudarthrobacter*, its stable association with Root TP, Stem TN, and Leaf TK suggests that it may participate in the nutrient-linked microbial processes underlying A54-mediated drought alleviation. This result indicates that the action of A54 was not a general enhancement of microbial abundance, but a shift of the rhizosphere toward a more stable and functionally coordinated state [27]. From the standpoint of innovation, this study did not analyze persistent taxa, Zi–Pi roles, and key taxa as separate pieces of evidence, but interpreted them together as manifestations of an A54-mediated selective assembly process.

### 3.5. Soil Functional Shifts Associated with Selective Microbial Assembly

Figure 5 supports this interpretation at the soil functional level. Under moderate and severe drought, A54-treated soils maintained higher levels of AN, AP, AK, S-Acp, S-Cat, S-Sc, and S-Ue. These variables did not change independently, but formed coordinated relationships, indicating that A54 altered an integrated soil functional environment rather than a few isolated soil indices. Mantel analysis further showed that overall bacterial community variation was associated with multiple soil functional variables, and that key keystone taxa were even more closely linked to these nutrient and enzyme indicators. Previous studies have shown that soil enzyme activity and available nutrient levels are important functional variables linking microbial processes to plant nutrient acquisition [28,29,30]. Therefore, the effect of A54 in this study was reflected not only in soil improvement, but also in the reorganization of the relationship between soil function and microbial assembly. In other words, A54 changed a rhizosphere functional background favorable for nutrient turnover and the maintenance of key taxa. In terms of innovation, this makes the present study different from many PGPR studies that only compare soil properties or only describe microbial composition, because the emphasis here is on the coupling between soil function and selective microbial response.

### 3.6. Molecular Support and Integrated Host–Soil–Microbe Responses

Figure 6 adds the final layer of molecular support. The A54 genome contained multiple functional modules related to nitrogen metabolism, ACC deaminase-associated ethylene regulation, IAA-related traits, oxidative defense, and osmotic or ion homeostasis. These functions were highly consistent with the plant physiological and soil responses observed in the greenhouse experiment, indicating that the effect of A54 was not only empirical, but also had a clear functional basis [18]. More importantly, representative genes showed differential expression across drought gradients, and *pyk*, *nifU*, *SOD2*, *catB*, *acds*, and *kdpA* were particularly responsive. This suggests that the drought response of A54 was not driven by a single pathway, but more likely involved coordinated regulation across central metabolism, nitrogen metabolism, oxidative defense, and ion regulation [31]. The novelty of this part lies in the fact that the study did not stop at plant phenotype or rhizosphere ecology, but further linked ecological and physiological results with bacterial functional potential and the expression of representative genes, thereby providing molecular support for the drought-alleviating role of A54.

Figure 7 finally integrates the preceding results into a continuous response network. Plant nutrient status showed systematic Spearman correlations with host growth, soil function, Shannon diversity, and key bacterial taxa. Among these variables, Stem TN and Leaf TK showed the broadest positive correlations, indicating that they occupied central positions in the A54 response network. At the same time, soil functional indices such as AN, AP, AK, and S-Sc were closely linked to these nutrient variables, and Pseudarthrobacter also showed positive correlations with several of them. This pattern supports the view that A54-mediated drought alleviation is associated with coordinated changes in plant nutrient maintenance, soil functional improvement, and selective microbial responses. The role of Figure 7 is therefore to provide an integrative correlation-based framework that links the results from Figure 1, Figure 2, Figure 3, Figure 4, Figure 5 and Figure 6, rather than direct causal proof of the underlying mechanisms. From the perspective of innovation, this nutrient-centered integration of host, soil, and key bacterial taxa substantially strengthens the mechanistic depth of the study and avoids a vague description of PGPR-mediated drought alleviation [14].

### 3.7. Limitations and Future Perspectives

Despite the relatively complete evidence chain obtained in this study, several limitations should be acknowledged. First, the experiment was conducted under greenhouse pot conditions with a single host species, one soil system, and one bacterial strain, and therefore, the stability and practical performance of A54 under field conditions still need to be tested. We also acknowledge that retaining 3–5 seedlings per pot may introduce additional variability in plant density. In addition, the relatively low number of biological replicates, particularly for physiological traits and qRT-PCR analyses, limits statistical power. These results should therefore be interpreted as indicative or exploratory rather than fully confirmatory. Second, the qRT-PCR results reflected gene expression responses of the strain under drought-related conditions, but they cannot yet represent in situ expression dynamics in the real rhizosphere. Future work should combine field inoculation, absolute quantification of key taxa, targeted metabolite measurements, and functional loss approaches to further verify the actual roles of ACC deaminase, potassium maintenance, and key taxa in A54-mediated drought alleviation.

### 3.8. Concluding Interpretation

Taken together, the effect of A54 on drought response in *Pinus sylvestris* var. *mongolica* seedlings was not driven by a single factor, but by the combined action of strain-level osmotic stress tolerance, improved host physiological status, maintenance of key nutrient traits, selective rhizosphere reassembly, and soil functional adjustment. In particular, Stem TN and Leaf TK showed strong central features in the integrative analysis, while the stable associations of *Pseudarthrobacter* with multiple nutrient and soil functional variables further suggest that specific key taxa may participate in A54-mediated drought regulation. Therefore, the role of A54 is better interpreted as a coordinated process across the host, soil, and microbial compartments, rather than as a simple growth-promoting effect or an isolated community shift. This perspective helps deepen the mechanistic understanding of PGPR-mediated drought responses in conifer seedlings.

## 4. Materials and Methods

### 4.1. Bacterial Strain, Plant Material, and Growth Substrate

*Pseudomonas chlororaphis* A54 (MT 280204) was isolated from the rhizosphere soil of *Pinus sylvestris* var. *mongolica* collected from the Zhanggutai Experimental Forest Farm, Liaoning Province, China. The strain was identified based on 16S rRNA gene sequencing and phylogenetic analysis, and is currently preserved in the Forest Microbiology Laboratory, Northeast Forestry University, Harbin, China. Seeds of *Pinus sylvestris* var. *mongolica* were obtained from the Zhanggutai nursery base, China, in the same year that the experiment was conducted. Before sowing, the seeds were surface-sterilized with 0.5% KMnO_4_ for 15 min, rinsed thoroughly with sterile water, germinated, and then sown in pots. The potting substrate consisted of peat, vermiculite, and sand mixed at a volume ratio of 2:1:1. Seeds were sown in plastic nursery pots measuring 18 cm × 18 cm × 16 cm, with an approximate volume of 4 L, and seedlings were thinned to 3-5 uniform plants per pot before inoculation and drought treatment.

### 4.2. In Vitro Characterization of Strain A54

The drought tolerance and plant growth-promoting traits of strain A54 were evaluated before the pot experiment. For osmotic stress tolerance, polyethylene glycol 6000 (PEG 6000, CAS No.25322-68-3, MedChem Express) was added to tryptone soy broth (TSB, CAT No.HY-157362, MedChem Express) to establish defined osmotic stress levels corresponding to 0, −0.05, and −0.30 MPa, according to the standard relationship between PEG concentration and osmotic potential. This assay was used as an in vitro approach to compare the osmotic stress tolerance of strain A54 under controlled water-potential conditions. These PEG-based osmotic stress levels were selected as representative strain-level stress gradients and were not intended to directly correspond to the ND, LD, MD, and SD treatments used in the greenhouse pot experiment. For biomass determination, the samples were first heated at 105 °C for a short period as an initial dehydration step to rapidly remove free moisture, and were then dried at 75 °C to constant weight before weighing. Each treatment was performed in triplicate.

ACC deaminase activity was determined using Dworkin–Foster (DF) medium supplemented with 3 mM ACC as the sole nitrogen source. Briefly, bacterial suspensions were first inoculated onto DF solid medium and incubated at 30 °C for 3 d. The strain was then transferred to TSB and cultured to the stationary phase. After collection by centrifugation, the cells were washed twice with 0.1 M Tris–HCl (pH 7.5), resuspended in DF liquid medium containing 3 mM ACC, and incubated at 28 °C with shaking at 250 rpm for 48 h. PEG 6000 was used to establish different osmotic potentials, corresponding to 0 MPa, −0.05 MPa, and −0.30 MPa. ACC deaminase activity was quantified based on α-ketobutyrate production. ACC deaminase activity was determined by measuring the amount of α-ketobutyrate produced from ACC. Quantification was based on an α-ketobutyrate standard curve, and enzyme activity was expressed as μmol α-ketobutyrate mg^−1^ protein h^−1^. PEG 6000 was included in this assay to impose osmotic stress and to evaluate whether ACC deaminase activity could be maintained under water-deficit-related conditions.

The plant growth-promoting traits of strain A54, including nitrogen fixation, phosphate solubilization, IAA production, siderophore production, and ACC deaminase activity, were evaluated using standard microbiological and biochemical assays. Nitrogen fixation was assessed on nitrogen-free medium, phosphate solubilization was determined on phosphate-solubilizing medium, IAA production was measured colorimetrically after growth in tryptophan-amended medium, siderophore production was evaluated on CAS medium, and ACC deaminase activity was determined based on α-ketobutyrate production from ACC [32]. These in vitro data are presented in the Supplementary Material Table S1.

### 4.3. Pot Experiment and Inoculation Design

A54 was cultured in nutrient broth at 30 °C for 24 h with shaking at 180 rpm. Bacterial cells were collected by centrifugation, resuspended in sterile water, and adjusted to OD_600_ = 0.6 (corresponding to approximately 1 × 10^8^ CFU mL^−1^). The bacterial suspension of strain A54 was applied directly to the rhizosphere zone of the seedlings during inoculation. The growth substrate was sterilized before use. No surfactant was added during the inoculation process. To maintain the presence and activity of strain A54 during the experimental period, each inoculated pot received 100 mL of bacterial suspension every 15 d, whereas the control pots received the same volume of sterile nutrient broth. The bacterial suspension was prepared by centrifugation and resuspension of the cells in sterile nutrient broth, so that the control and inoculated treatments received the same carrier solution.

The experiment followed a 2 × 4 factorial design with two inoculation treatments (CK and A54) and four drought gradients: normal water supply (ND, approximately 80% relative soil water content), light drought (LD, approximately 60% relative soil water content), moderate drought (MD, approximately 45% relative soil water content), and severe drought (SD, approximately 30% relative soil water content). Each treatment combination initially contained ten pots in the greenhouse experiment. After thinning, 3–5 seedlings were retained in each pot. The pot was treated as the experimental unit throughout the study. For growth, plant nutrient, physiological, qRT-PCR, and related measurements, three pots were randomly selected from each treatment for sampling. Within each selected pot, sampled seedlings were measured and their values were averaged to obtain one pot-level value before statistical analysis. Soil water content was monitored gravimetrically and maintained within the target range throughout the experiment. Drought treatment lasted for 45 d, after which plant and rhizosphere soil samples were collected for subsequent analyses.

### 4.4. Measurement of Seedling Growth and Plant Nutrient Contents

After three months of water stress, plants were randomly selected to measure their seedling height, diameter, root fresh weight, and shoot fresh weight. Shoot and root samples were heated at 105 °C for 15 min and then dried at 75 °C to constant weight to determine shoot dry weight and root dry weight. For nutrient analysis, root, stem, and leaf tissues were sampled separately, oven-dried, finely ground, and analyzed for organic matter (OM), total nitrogen (TN), total phosphorus (TP), and total potassium (TK) following Cui et al. (2018) [33]. Plant OM was determined by the potassium dichromate volumetric method with external heating (Walkley–Black method). Plant samples for TN, TP, and TK determination were first digested with sulfuric acid and hydrogen peroxide. TN was determined by the Kjeldahl method or flow injection analysis, TP was measured by spectrophotometry, and TK was determined by flame photometry or atomic absorption spectrometry. Quantification was performed using standard solutions and standard curves.

### 4.5. Determination of Plant Physiological Traits

Fresh plant tissues were immediately frozen in liquid nitrogen and ground into powder. The activities of superoxide dismutase (SOD, product no. A001), peroxidase (POD, product no. A084), and catalase (CAT, product no. A007), together with the contents of malondialdehyde (MDA, product no. A003), proline (PRO, product no. A107), and abscisic acid (ABA, product no. H251), were measured using commercial kits from Nanjing Jiancheng Bioengineering Institute (Jiangsu, China) according to the manufacturer’s instructions. Ethylene (ET) content was determined using a commercial assay kit according to the manufacturer’s instructions and was used as a relative physiological indicator for comparison among treatments under the same experimental conditions. These indices were used to evaluate antioxidant defense, osmotic regulation, membrane lipid peroxidation, and hormonal responses under drought stress. For the physiological assays based on commercial kits, approximately 100 mg of fresh tissue was used for each extraction. All enzyme activities and metabolite contents were normalized on a fresh-weight basis (g^−1^ FW). Three biological replicates were used for physiological measurements.

### 4.6. Determination of Soil Physicochemical Properties and Enzyme Activities

Rhizosphere soil was operationally defined as the soil tightly adhering to the roots after gentle shaking to remove loosely attached soil. The remaining root-adhering soil was then collected by brushing and passed through a 20-mesh sieve before subsequent analyses. Soil OM, TN, TP, TK, available nitrogen (AN), available phosphorus (AP), and available potassium (AK) were determined following Cui et al. (2018) [33]. Soil acid phosphatase (S-Acp, product no. A060), catalase (S-Cat, product no. A007), sucrase (S-Sc, product no. A082), and urease (S-Ue, product no. A121) were measured using commercial kits from Nanjing Jiancheng Bioengineering Institute (Nanjing, China) according to the manufacturer’s instructions. For soil enzyme assays, 1.0 g of fresh soil passed through a 2 mm sieve was used for each measurement. The corresponding extraction and reaction solutions were added according to the kit instructions for incubation and colorimetric determination. All soil physicochemical indices and enzyme activities were calculated on a dry soil weight basis. Soil organic matter (OM) was expressed as g·kg^−1^; AN, AP, and AK were expressed as mg·kg^−1^; and soil acid phosphatase (S-Acp), catalase (S-Cat), sucrase (S-Sc), and urease (S-Ue) activities were expressed as U·g^−1^. Three biological replicates were analyzed for each treatment.

### 4.7. Rhizosphere Bacterial Community Sequencing and Bioinformatics Analysis

Genomic DNA was extracted using the EZNA Soil DNA Kit (Omega Bio-TEK, Norcross, GA, USA). The PCR products were purified using the AxyPrep DNA Gel Extraction Kit (Axygen Biosciences, Union City, CA, USA), and DNA concentration was quantified using QuantiFluor-ST (Promega, Madison, WI, USA). Bacterial community sequencing was performed by Majorbio Bio-Pharm Technology Co., Ltd. (Shanghai, China) on the Illumina MiSeq platform. The bacterial 16S rRNA gene was amplified using primers 338F (5′-ACTCCTACGGGAGGCAGCAG-3′) and 806R (5′-GGACTACHVGGGTWTCTAAT-3′). Raw reads were merged and quality-filtered using FLASH (v1.2.11) and fastp (v0.19.6) [34,35]. The amplicon data generated in this study were deposited in the NCBI Sequence Read Archive under accession number PRJNA774501.

Raw reads were processed using the standard amplicon sequence analysis pipeline, and sequences were clustered into OTUs at 97% similarity. Taxonomic assignment was performed against the SILVA 128 16S bacterial database using a classification confidence threshold of 0.7. Alpha-diversity indices, including Chao richness and Shannon diversity, were calculated after normalization. Community structure was evaluated by principal component analysis (PCA), and group differences were further tested by analysis of similarities (ANOSIM). Relative abundances at the phylum and genus levels were summarized for taxonomic composition analysis. Differential taxa among treatments were identified using LEfSe. Relationships between bacterial community composition and environmental variables were examined by redundancy analysis (RDA) and Mantel tests. Functional profiles inferred from 16S amplicon data and the relative contributions of environmental variables were further used to interpret potential ecological shifts in the rhizosphere bacterial community.

### 4.8. Genome Sequencing, Annotation, and qRT-PCR Validation

A54 was cultured in nutrient broth at 28 °C with shaking at 180 rpm, and bacterial cells were harvested for genomic DNA extraction and whole-genome sequencing. Genome assembly and quality control were performed using SOAP denovo (version 2.0) [36], Unicycler (version 0.10) [37], and SMRT (version 2.3.0) [38] workflows as appropriate. Genome assembly was conducted using Unicycler, and a single-platform assembly strategy was applied according to the sequencing data generated in this study. The resulting genome was subsequently used for downstream functional annotation. Transfer RNA and ribosomal RNA genes were identified using tRNAscan-SE and Barrnap, respectively. Functional annotation was performed against the Gene Ontology (GO), Clusters of Orthologous Groups (COG), and Kyoto Encyclopedia of Genes and Genomes (KEGG) databases. The genome sequence data were deposited in the NCBI Sequence Read Archive under accession number PRJNA730979.

Total RNA was extracted from strain A54 using the Bead/SDS/phenol method and first-strand cDNA was synthesized using a first-strand cDNA synthesis kit. qRT-PCR was performed on a Rotor-Gene 6000 instrument (QIAGEN, Hilden, Germany) using SYBR Premix Ex Taq chemistry in a 25 μL reaction system. Each treatment included two biological replicates and three technical replicates. Relative expression levels were calculated using the 2^−ΔΔCt^ method with gyrB as the reference gene. The genes analyzed were pyruvate kinase (pyk), nitrogen fusion protein (nifU), superoxide dismutase (SOD2), catalase (catB), 1-aminocyclopropane-1-carboxylic acid deaminase (acds), potassium-transporting ATPase potassium-binding subunit (kdpA), proline dehydrogenase (putA), and tryptophan 2-monooxygenase (iaaM). Primer sequences are provided in Supplementary Table S7.

### 4.9. Statistical Analysis

Plant growth, plant nutrient, physiological, and soil data were analyzed according to the 2 × 4 factorial design, with inoculation and drought gradient as fixed factors. Statistical analyses were conducted using the SPSS software package (Version 19.0). Two-way analysis of variance (ANOVA) was used to test the main effects of inoculation, drought, and their interaction, followed by Duncan’s multiple range test at *p* < 0.05. Data are presented as mean ± standard error. Before ANOVA, the data were evaluated for normality and homogeneity of variance using the Shapiro–Wilk test and Levene’s test, respectively. Statistical analyses were then performed according to the experimental design described in this study. Correlation analysis was performed using Spearman correlation, and the resulting *p*-values were corrected for multiple testing using the false discovery rate (FDR) method. Alpha-diversity analysis was conducted using Mothur (Version 1.30.2) [39], and multivariate analyses, including PCA, ANOSIM, RDA, Mantel tests, LEfSe-related analyses, and graphical visualization, were performed in R (Version 4.5.3) [40]. Beta-diversity analysis was conducted using Bray–Curtis distance. ANOSIM was performed with 999 permutations. RDA was conducted using correlation biplot scaling. Differential taxa were identified using LEfSe with the ALL-against-all multi-class comparison strategy and an LDA threshold of 3.

## 5. Conclusions

In summary, *Pseudomonas chlororaphis* A54 improved drought tolerance in *Pinus sylvestris* var. *mongolica* seedlings through coordinated changes in host nutrient and physiological status, rhizosphere bacterial assembly, and soil functional variables. Stem TN and Leaf TK emerged as important nutrient traits associated with this response, while *Pseudarthrobacter* was identified as a potentially relevant bacterial taxon within the inoculated rhizosphere system. These findings support the potential application of A54 as a bio-inoculant for drought-prone afforestation systems. Future studies should further validate its performance and stability under field conditions. Future studies should further validate the performance and stability of A54 under field conditions and assess its application potential in broader conifer afforestation systems.

## Figures and Tables

**Table 1:** Effect of A54 strain inoculation on the growth of *P. sylvestris* var. *mongolica* seedlings under different gradients.

Drought Gradient	Treatments	Seedling Height (cm)	Diameter (mm)	Shoot Fresh Weight (g)	Shoot Dry Weight (g)	Root Fresh Weight (g)	Root Dry Weight (g)
ND	CK	7.75 ± 0.79 d	1.09 ± 0.01 a	0.32 ± 0.00 c	0.27 ± 0.01 a	0.10 ± 0.00 c	0.06 ± 0.00 a
	A54	13.84 ± 1.12 a	1.12 ± 0.10 a	0.51 ± 0.03 a	0.28 ± 0.03 a	0.16 ± 0.00 a	0.06 ± 0.00 a
LD	CK	6.91 ± 0.35 d	0.90 ± 0.05 b	0.31 ± 0.01 c	0.22 ± 0.02 b	0.09 ± 0.00 c	0.05 ± 0.00 b
	A54	11.34 ± 0.50 b	0.97 ± 0.17 b	0.54 ± 0.00 a	0.25 ± 0.00 a	0.15 ± 0.00 a	0.06 ± 0.00 a
MD	CK	6.80 ± 0.34 d	0.83 ± 0.09 c	0.29 ± 0.02 c	0.17 ± 0.00 c	0.08 ± 0.00 d	0.04 ± 0.00 c
	A54	11.54 ± 2.01 b	1.07 ± 0.09 a	0.42 ± 0.04 b	0.28 ± 0.00 a	0.13 ± 0.01 b	0.05 ± 0.00 b
SD	CK	4.81 ± 0.98 e	0.65 ± 0.11 d	0.25 ± 0.01 d	0.14 ± 0.01 d	0.06 ± 0.00 e	0.03 ± 0.00 d
	A54	9.03 ± 1.00 c	0.90 ± 0.09 b	0.45 ± 0.01 b	0.20 ± 0.00 b	0.10 ± 0.00 c	0.05 ± 0.00 b

The results are expressed as mean ± standard error (*n* = 3 pot-level biological replicates), and different letters (a–e) indicate significant differences between treatments (*p* < 0.05).

## Data Availability

The amplicon data generated in this study were deposited in the NCBI Sequence Read Archive under accession number PRJNA774501. The genome sequence data were deposited in the NCBI Sequence Read Archive under accession number PRJNA730979.

## Supplementary Materials

The following supporting information can be downloaded at: https://www.mdpi.com/article/10.3390/plants15101503/s1, Figure S1: In vitro drought tolerance and ACC deaminase activity of strain A54 under different osmotic stress levels; Table S1: Plant growth promoting traits of A54 strain; Table S2: ANOVA results showing the effects of strain A54 inoculation, drought gradients, and their interaction on plant antioxidant enzymes, osmotic regulators, membrane lipid peroxidation products, and phytohormones; Table S3: Community diversity indexes of each treatment group under different drought gradients; Table S4: Monte Carlo experiment on the effects of soil nutrients and enzyme activity indicators onbacterial community structure; Table S5: Genome Assembly Statistics of A54 Strain; Table S6: Full list of annotated genes in strain A54 potentially related to plant growth promotion and drought adaptation; Table S7: PCR primers for PGP and drought resistance response testing genes.

## Abbreviations

The following abbreviations are used in this manuscript:

PGPR	plant growth-promoting rhizobacteria
PEG	polyethylene glycol
NCBI	National Center for Biotechnology Information
ROS	reactive oxygen species
CK	uninoculated control
ND	normal water supply
LD	light drought
MD	moderate drought
SD	severe drought
DF	Dworkin–Foster medium
TSB	tryptic soy broth
DNA	deoxyribonucleic acid
ELISA	enzyme-linked immunosorbent assay
ABA	abscisic acid
ACC	1-aminocyclopropane-1-carboxylate
TN	total nitrogen
TP	total phosphorus
TK	total potassium
OM	organic matter
AN	available nitrogen
AP	available phosphorus
AK	available potassium
ANOSIM	analysis of similarities
CAT	catalase
cDNA	complementary DNA
ET	ethylene
MDA	malondialdehyde
S-Acp	soil acid phosphatase
S-Cat	soil catalase
S-Sc	soil sucrase
S-Ue	soil urease
SOD	superoxide dismutase
POD	peroxidase
PRO	proline
OTU	operational taxonomic unit
PCA	principal component analysis
RDA	redundancy analysis
SMRT	single-molecule real-time
RNA	ribonucleic acid
qRT-PCR	quantitative reverse transcription polymerase chain reaction
IAA	Indole-3-acetic acid
GO	Gene Ontology
COG	Clusters of Orthologous Group
KEGG	Kyoto Encyclopedia of Genes and Genome
