# Supplementary material for: Pseudomonas chlororaphis A54 Enhances Drought Tolerance in Pinus sylvestris var. mongolica Through Coordinated Plant Physiological, Rhizosphere Microbial, and Soil Functional Responses
Source: Plants (Basel). 2026 May 14;15(10):1503. doi: 10.3390/plants15101503 (PMC13210770; doi:10.3390/plants15101503)
Supplement: Supplementary file 1 [file plants-15-01503-s001.zip › plants-4274235-supplementary.pdf]

Supplementary materials

Table S1. Plant growth promoting traits of A54 strain.

PGP	Treatments A54
N-fixation Yield (mg/L)	32.32 ± 3.60
P content (ug/mL)	13.25 ± 3.89
IAA Yield (ug/mL)	4.71 ± 0.78
Siderophores production	+
HCN	+
Pectinase	+
Chitinase	+
Cellulase	+
Protease	+

(−) not detected; (+) production. Different letters (a, b, c) indicate significant differences between treatments (*P*<0.05).

Table S2. ANOVA results showing the effects of strain A54 inoculation, drought gradients, and their interaction on plant antioxidant enzymes, osmotic regulators, membrane lipid peroxidation products, and phytohormones.

Variables		SOD	CAT	POD	PRO	MDA	ET	ABA
A54 (ND)	F	103.22	54.57	79.83	132.29	59.88	69.46	63.82
	Sig.	***	***	***	***	***	***	***
Drought (LD)	F	79.54	1.97	1.42	2.00	9.86	1.07	3.57
	Sig.	***	0.13ns	0.32ns	0.55ns	**	0.17ns	*
Drought (MD)	F	99.47	24.36	10.12	8.97	9.16	29.43	38.66
	Sig.	***	***	**	**	**	***	***
Drought (SD)	F	162.51	18.62	17.05	63.26	34.59	38.38	74.56
	Sig.	***	***	***	***	***	***	***
A54×Drought (LD)	F	97.77	3.42	19.95	107.46	23.27	1.99	6.45
	Sig.	***	*	***	***	**	0.20ns	**
A54×Drought (MD)	F	75.45	24.56	28.74	48.96	5.44	19.20	35.31
	Sig.	***	***	***	***	*	**	***
A54×Drought (SD)	F	87.54	17.66	23.24	61.28	46.62	39.27	94.27
	Sig.	***	**	***	***	***	***	***

ns, not significant; **P* < 0.05; ** *P* < 0.01; *** *P* < 0.001.*

Table S3. Community diversity indexes of each treatment group under different drought gradients.

Drought gradient	Treatments	Coverage	Chao	Shannon
ND	CK	0.9931 ± 0.01 a	1952.44 ± 64.79 b	5.22 ± 0.04 b
	A54	0.9924 ± 0.00 a	2229.53 ± 83.75 a	5.48 ± 0.04 a
LD	CK	0.9930 ± 0.01 a	1736.75 ± 62.56 c	5.06 ± 0.08 b
	A54	0.9927 ± 0.00 a	1927.31 ± 49.00 b	5.52 ± 0.05 a
MD	CK	0.9928 ± 0.00 a	1607.79 ± 89.36 d	4.92 ± 0.02 d
	A54	0.9931 ± 0.01 a	1908.39 ± 57.57 b	5.41 ± 0.01 ab

SD	CK	0.9930 ± 0.01 a	1642.94 ± 58.90 d	5.02 ± 0.05 c
	A54	0.9916 ± 0.00 a	1905.65 ± 124.20 b	5.52 ± 0.04 a

The results are expressed as mean ± standard error (n = 3), and different letters (a, b, c, d) indicate significant differences between treatments (P<0.05).

Table S4. Monte Carlo experiment on the effects of soil nutrients and enzyme activity indicators on

bacterial community structure.

**Variables**	**R2**	**P**
OM	0.4991	0.004**
TN	0.1382	0.220
AN	0.8342	0.001***
TP	0.5337	0.002**
AP	0.5221	0.004**
TK	0.3924	0.009**
AK	0.1735	0.138
S-Ue	0.7824	0.001***
S-Sc	0.6487	0.001***
S-Cat	0.7100	0.001***
S-Acp	0.8937	0.001***

Table S5. Genome Assembly Statistics of A54 Strain.

**Category**	**A54**
Genome size（bp）	6489442
Gene average size（bp）	987
N（%）	0. 12
GC（%）	56.97
Q20（%）	97.25
Q30（%）	91.04
Gene num	5745
Protein-coding genes	5654
t RNA	62
S rRNA	4
Protein with signal peptides	838
Genes assigned to COGs	5321
Genes assigned to GOs	6610
Genes assigned to KEGGs	5521

Table S6. Full list of annotated genes in strain A54 potentially related to plant growth promotion and drought adaptation.

**Gene**	**Ec No.**	**Category**
treS	EC:5.4.99.1	Starch and sucrose metabolism
bglX	EC:3.2.1.21	Starch and sucrose metabolism
treZ	EC:3.2.1.141	Biosynthesis of secondary metabolites
lip	EC:3.1.1.3	Glycerolipid metabolism
SOD2	EC:1.15.1.1	Peroxisome
catB	EC:1.11.1.6	Tryptophan metabolism
katG	EC:1.11.1.21	Tryptophan metabolism
katG	EC:1.11.1.21	Phenylalanine metabolism
gabD	EC:1.2.1.16	Alanine, aspartate and glutamate metabolism

davT	EC:2.6.1.48	Lysine degradation
kdpD	EC:2.7.13.3	Two-component system
putA	EC:1.5.5.2	Biosynthesis of antibiotics
proV	EC:7.6.2.9	ABC transporters
proW	-	Membrane transport
proX	-	Membrane transport
xdhA	EC:1.17.1.4	Purine metabolism
betA	EC:1.1.99.1	Glycine, serine and threonine metabolism
betB	EC:1.2.1.8	Glycine, serine and threonine metabolism
acds	EC:3.5.99.7	Cysteine and methionine metabolism
gdh	EC:1.4.1.2	Alanine, aspartate and glutamate metabolism
nhaA	-	Energy Metabolism
atpA	EC:7.1.2.2	Energy metabolism
xdhA	EC:1.17.1.4	Nucleotide metabolism
BADH	EC:1.1.1.31	Amino acid metabolism
gor	EC:1.8.1.7	Metabolism of other amino acids
pyk	EC:2.7.1.40	Glycolysis/Gluconeogenesis
aceB	EC:2.3.3.9	Pyruvate metabolism
ppc	EC:4.1.1.31	Carbon metabolism
pycA	EC:6.4.1.1	Citrate cycle (TCA cycle)
gltA	EC:2.3.3.1	Glyoxylate metabolism
aroK	EC:2.7.1.71	Phenylalanine, tyrosine and tryptophan biosynthesis
pstS	-	ABC transporters
pstA	-	ABC transporters
pstB	EC:7.3.2.1	ABC transporters
GABA	EC:6.3.1.2	Nervous system
nifU	-	Nitrogen metabolism
nasA	EC:1.7.99.-	Nitrogen metabolism
NRT	-	Nitrogen metabolism
nirB	EC:1.7.1.15	Nitrogen metabolism
nirD	EC:1.7.1.15	Nitrogen metabolism
cynT	EC:4.2.1.1	Nitrogen metabolism
glnA	EC:6.3.1.2	Nitrogen metabolism
ncd2	EC:1.13.12.16	Nitrogen metabolism
gltD	EC:1.4.1.13	Nitrogen metabolism
gltB	EC:1.4.1.13	Nitrogen metabolism
ntrB	EC:2.7.13.3	Two-component system
arcC	EC:2.7.2.2	Nitrogen metabolism
cynS	EC:4.2.1.104	Nitrogen metabolism
kdpA	-	Environmental Information Processing
kdpE	-	Environmental Information Processing
kdpD	EC:2.7.13.3	Environmental Information Processing
kdpC	-	Environmental Information Processing
kdpB	EC:7.2.2.6	Environmental Information Processing
iaaM	EC:1.13.12.3	NAD(P)/FAD-dependent oxidoreductase
atoB	EC:2.3.1.9	Tryptophan metabolism
pdhD	EC:1.8.1.4	Tryptophan metabolism
echA	EC:4.2.1.17	Tryptophan metabolism
amiE	EC:3.5.1.4	Tryptophan metabolism
gcdH	EC:1.3.8.6	Tryptophan metabolism
hemH	EC:4.99.1.1	Porphyrin and chlorophyll metabolism
afuA	-	ABC transporters
afuB	-	ABC transporters
afuC	EC:7.2.2.7	ABC transporters
bfr	EC:1.16.3.1	Porphyrin and chlorophyll metabolism
hemE	EC:4.1.1.37	Porphyrin and chlorophyll metabolism
hemO	EC:1.14.99.58	Porphyrin and chlorophyll metabolism
hemB	EC:4.2.1.24	Porphyrin and chlorophyll metabolism

Table S7. PCR primers for PGP and drought resistance response testing genes.

Gene	Primers	Sequence(5′to3′)
pyk	pyk-F<br>pyk-R	CGCAGCCGTTCATTTAGCA<br>TTGTTCTCGTTCCCGTTCC
nifU	nifU-F<br>nifU-R	CGCGGATCCATGGATTTCTTTGCC<br>CGGCTCGAGTTATGTATCTTTGTTT
catB	catB-F<br>catB-R	GACAAGGAGAACAATTTCCAACAG<br>AGTAGGAGATCCAGATGCCAC
acds	ACC1140<br>ACC1623	GCTGGTGCAGGAAAACTGGG<br>GAARCGCGTRTCGAGCACCAC
SOD2	SOD2-F<br>SOD2-R	TCGTCCTCCTCATCCTCCTC<br>AGACACTTCCAGTGACAGTG
kdpA	kdpA-in-F<br>kdpA-in-R	GAGGGCCTAAACGATGTCGTGGAGCAAA<br>GCGTGCCACCGACATCCCCAGACCGA
putA	pu<br>pd	CTCTAGAGTCAGCCGATCGCCATCAG<br>CGAGCTCGATGAGCCAGACCAGCTTC
iaam	iaaM F<br>iaaM R	CTTACGAGAAAGGCACGAC<br>TAGATGCTGGGCAAACG
gyrB	gyrB-F<br>gyrB-R	GTCGTGCGTATCGTGAGCGT<br>TGCTGCCGTTGTACACGATT

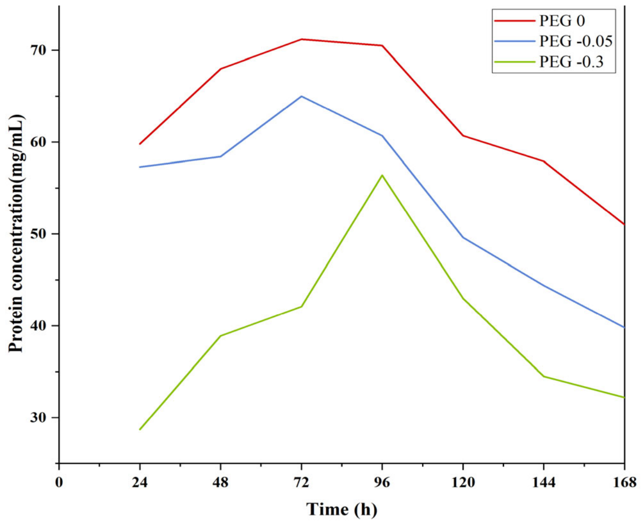

(a)

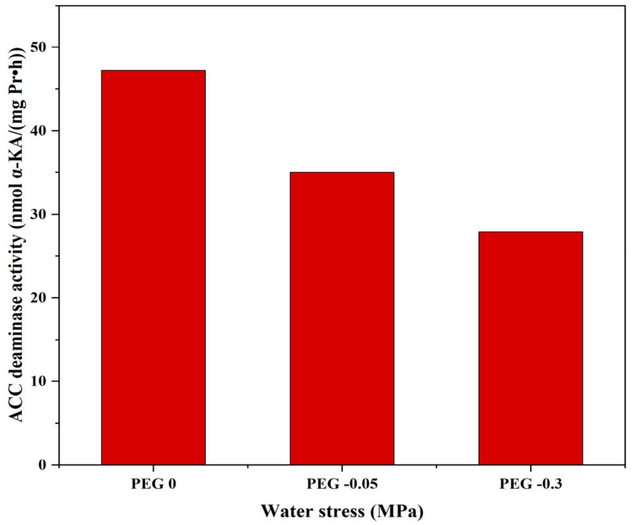

(b)

Figure S1. In vitro drought tolerance and ACC deaminase activity of strain A54 under different osmotic stress levels. **(a)** Growth curve of strain A54 under different water potentials, expressed as protein concentration over time. **(b)** ACC deaminase activity of strain A54 under different water stress levels. PEG 0, no osmotic stress; PEG -0.05 and PEG -0.3, reduced water potential treatments. Values are presented under different osmotic stress conditions.
